# Concanavalin A Toxicity Towards Potato Psyllid and Apoptosis Induction in Midgut Cells

**DOI:** 10.3390/insects11040243

**Published:** 2020-04-14

**Authors:** Xiao-Tian Tang, Freddy Ibanez, Cecilia Tamborindeguy

**Affiliations:** Department of Entomology, Texas A&M University, College Station, TX 77843, USA; tangxt@tamu.edu (X.-T.T.); fibanezcarrasco@ufl.edu (F.I.)

**Keywords:** concanavalin A, lectin, toxicity, potato psyllid, midgut, apoptosis

## Abstract

Concanavalin A (ConA), a legume lectin, has been drawing increasing attention in recent years concerning its toxicity against insects and its potential application in pest management. In an attempt to evaluate the effect of ConA on potato psyllid (*Bactericera cockerelli*), an economically important pest of solanaceous crops, the effect of ConA on potato psyllid survival, psyllid gut nuclear morphology, and expression of psyllid caspase genes were evaluated. Our results determined that artificial diet-feeding assays using ConA had deleterious effects on potato psyllids, resulting in significant psyllid mortality following ingestion. We also found that an apoptotic response was induced by ConA in psyllid midgut cells, which was demonstrated by the DNA fragmentation and abnormal nuclear architecture in the midgut cells. Following ConA ingestion, there was also upregulation of caspase genes in the psyllid midguts. Therefore, a key mechanism behind ConA toxicity towards potato psyllid probably involves the induction of apoptosis in midgut cells. This study could provide a better understanding of the mechanisms underlying ConA toxicity in insects and be a stepping stone towards the development of new psyllid control strategies based on plant lectins.

## 1. Introduction

Lectins are a class of plant proteins with at least one non-catalytic domain that can specifically and reversibly bind to carbohydrates without altering the covalent structure of the recognized glycosyl ligands [1,2]. Lectins with insecticidal properties against pests that could be used as defense molecules against insect herbivores and plant pathogens have been identified [3,4]. In particular, lectins show the greatest potential for exploitation in transgenic-based pest control strategies [5]. These proteins could be important as control agents for hemipteran pests because these insects are not susceptible to *Bacillus thuringiensis* (Bt) toxins [6,7].

Concanavalin A (ConA), a lectin from *Canavalia ensiformis*, is one of the most extensively investigated members of the lectin family of plant proteins [8]. ConA has previously been demonstrated to be detrimental to multiple pest species, such as the tomato moth, the cotton bollworm/legume pod borer, as well as several aphid species [9,10,11,12].

Despite the increasing interest in the insecticidal properties of plant lectins, the mechanisms behind their toxic properties in insects are not well understood. Lectin binding to insect proteins could be an essential step in exerting a toxic effect. Attempts have been made to identify lectin-binding receptors in insect guts and to determine the molecular basis of lectin toxicity to insects [13,14,15,16]. A majority of lectins that affect insects were shown to bind in areas of the midgut, the primary insecticide target tissue, and some lectins, such as *Galanthus nivalus* agglutinin (GNA) could also cross the gut epithelium reaching the hemolymph and other tissues [17,18]. In mammals, the mechanisms behind the toxic properties of lectins have been extensively studied, and were shown to involve the induction of programmed cell death, a cell immune process [19,20,21,22]. Indeed, some legume lectins such as *Lens culinaris* agglutinin (also known as LCA), ConA, and Phytohemagglutinin (PHA) are highly cytotoxic and can induce apoptosis of cancer cells [23]. Therefore, one key mechanism of lectin toxicity against insects could be associated with inducing cell death in insect midgut cells. However, the literature associated with apoptosis induction in hemipterans by lectins is still limited. Only two studies showed that ConA could induce cell apoptosis in the midgut of grain aphids and bird cherry-oat aphids [11,12].

Among hemipteran insects, the potato psyllid (also known as the tomato psyllid), *Bactericera cockerelli*, is a serious pest of solanaceous crops. This insect species is a phloem feeder that can directly affect plant growth, and also can transmit the phloem-limited bacterium, ‘*Candidatus* Liberibacter solanacearum’ (Lso) [24]. Presently, two Lso haplotypes (LsoA and LsoB) have been identified in North America. LsoA and LsoB can infect numerous solanaceous crops and cause extremely damaging diseases (e.g., zebra chip in potatoes). Currently, potato psyllid control relies on insecticide applications but even with conventional insecticides, this pest is difficult to manage [25]. Furthermore, commercially acceptable genetic resistance against the potato psyllid or Lso has not yet been identified in potato or other solanaceous crops [26]. Therefore, it is crucial to explore alternative strategies to control potato psyllids. ConA, a promising toxic agent against hemipteran insects, could be detrimental to potato psyllids. In the present study, we evaluated the toxic effect of ConA-containing artificial diets on potato psyllid survival. To assess if ConA induced apoptosis in the gut of potato psyllids, we examined the nuclear architecture of gut epithelial cells, performed TUNEL (terminal deoxynucleotidyl transferase dUTP nick end labeling) assays, and evaluated the expression of caspase genes after ConA-feeding. Intracellular bacteria such as Lso might be able to manipulate insect host responses, including programmed cell death [27]. Therefore, we also tested the effect of ConA on psyllids harboring LsoA and LsoB, on psyllids that did not harboring Lso to evaluate if the presence of the bacterial pathogen could protect the insects from ConA. This study may be a stepping stone to unravel the mechanisms behind ConA toxicity towards potato psyllids, and could provide valuable information for the use of plant lectins for pest management. Additionally, this study also provides knowledge about apoptosis in the psyllid gut in response to *Liberibacter* bacteria, which are intracellular pathogens.

## 2. Materials and Methods 

### 2.1. Insect

Lso-free, LsoA-, and LsoB-infected psyllid colonies [28] were maintained separately on tomato plants in insect cages (24 × 13.5 × 13.5 cm, BioQuip, Compton, CA, USA) at room temperature 24 ± 1 °C and photoperiod of 16:8 h (L:D). Young virgin female adults (1 to 3 days-old) were used for the artificial diet feeding assays, tissue observations, and gene expression analyses. 

### 2.2. Feeding Bioassays

**Figure 1:**
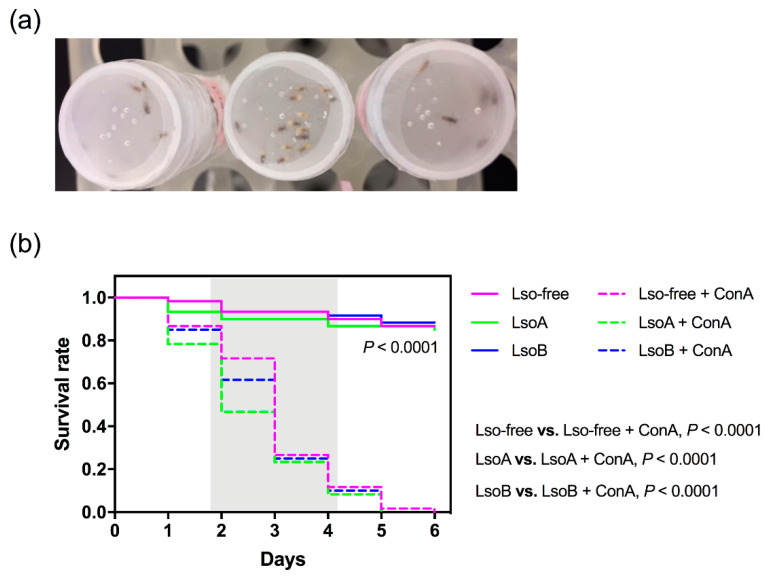
Feeding assays and Concanavalin A (ConA) toxicity towards potato psyllid. (**a**) Apparatus for feeding assays. The chambers were covered by two sheets of Parafilm with liquid diet between the two layers. (**b**) Mortality of *Candidatus* Liberibacter solanacearum (Lso)-free, LsoA-, and LsoB-infected potato psyllids following feeding on artificial diets without (control) and with ConA at the concentration of 2000 μg/mL. *p*-value refers to the log-rank test. The gray region indicates a significant mortality of psyllids after feeding ConA.

The liquid diet used for psyllid feeding bioassays was prepared with a sterilized solution of 15% (w:v) sucrose and 1× phosphate-buffered saline (1× PBS) solution (Sigma-Aldrich, St. Louis, MO, USA). Concanavalin A (MP Biomedicals, Solon, OH) was incorporated into the diet at a concentration of 2000 μg/mL [11]. Control diets (without ConA) were also included in the experiment. Young female adults from the Lso-free, LsoA- or LsoB-infected potato psyllid colonies were collected and placed in plastic feeding chambers (*h* = 2 cm, *Φ* = 3 cm). The chambers were covered by two sheets of Parafilm with 100 μL of the liquid diet described above in between the two layers (Figure 1a). The diet was replaced as required. Psyllid survival was monitored every 24 h. Three replicates consisting of 30 psyllid individuals each from the Lso-free, LsoA-, or LsoB-infected potato psyllid colonies were analyzed in the feeding assays.

### 2.3. Nuclear Architecture and TUNEL Assay of Gut Epithelial Cells

To investigate whether ConA impacted the nuclear architecture of the gut epithelial cells, Lso-free, LsoA-, or LsoB-infected potato psyllids were allowed to feed on diets containing 2000 μg/mL of ConA or control diet (without ConA) for 72 h. Subsequently, the psyllid guts were dissected in 1× PBS under the stereomicroscope (Olympus) and then fixed in 4% paraformaldehyde for 30 min at room temperature. After fixation, the guts were incubated with Sudan Black B (SBB) (Sigma-Aldrich, MO) for 20 min to quench autofluorescence [29]. Then, the guts were washed three times with 1× PBS containing 0.05% Tween 20 (PBST), mounted using Vectashield mounting medium with DAPI (Vector Laboratories Inc., Burlingame, CA, USA), covered with a glass coverslip and sealed with nail polish. At least 20 guts per treatment were examined using an Axioimager A1 microscope (Carl Zeiss microimaging, Thornwood, NY, USA). The images were collected and analyzed with the Axiovision Rel 4.8 software (Carl Zeiss, Göttingen, Germany).

To test DNA fragmentation of apoptotic cells, the guts from Lso-free, LsoA-, or LsoB-infected female potato psyllids were dissected in 1× PBS under the stereomicroscope (Olympus) and then fixed in 4% paraformaldehyde for 2 h at room temperature. The guts were blocked by 5% bovine serum albumin in 1× PBS with 0.1% Tween 20, then incubated with TUNEL for 6 h. TUNEL staining was performed using the In Situ Cell Death Detection Kit (Roche, Basel, Switzerland) [30]. After washing three times with PBS, samples were mounted using Vectashield mounting medium with DAPI, covered with a coverslip and sealed with nail polish. At least 20 guts per treatment were examined using Axioimager A1 microscope and analyzed as previously described.

### 2.4. Gene Expression of Caspases

The caspase genes involved in apoptosis pathways were identified by searching the psyllid transcriptome datasets [31] and verified by sequencing [32]. The primers for sequence validation can be found in Table S1. For the gene expression experiment, young female adults from the Lso-free, LsoA-, or LsoB-infected colonies were allowed to consume 2000 μg/mL ConA-containing diets for 72 h. Control treatments (without ConA) were also included. Three replicates were analyzed for each treatment, and each replicate had 30 psyllid individuals. After the exposure to ConA-containing diet, the psyllid guts were dissected under the stereomicroscope (Olympus) as previously described. RNA from a pool of 30 psyllids guts was purified using RNeasy Mini Kit (Qiagen, Hilden, Germany). The total RNA was reverse transcribed using Verso cDNA Synthesis kit (Thermo, Waltham, MA, USA) plus anchored-Oligo (dT) primers following the manufacturer’s instructions. Genomic DNA was eliminated by DNase I treatment with Turbo DNase (Ambion, Invitrogen, CA, USA). The expression of caspase genes was evaluated by quantitative PCR (qPCR). The gene-specific primers for qPCR can be found in Table S1. qPCR reactions were performed using SensiFAST SYBR Hi-ROX Kit (Bioline, Taunton, MA, USA) according to the manufacturer’s instructions. Each reaction contained 5 ng of cDNA, 250 nM of each primer (Table S1) and 1× of SYBR Green Master Mix; the volume was adjusted with nuclease-free water to 10 μL. The qPCR program was 95 °C for 2 min followed by 40 cycles of 95 °C for 5 sec and 60 °C for 30 sec. The qPCR assays were run using an Applied Biosystems QuantStudio 6 Flex Real-Time PCR System (Applied Biosystems). Reactions for all samples were performed in triplicates with a negative control (no cDNA) in each run. The relative expression of the candidate genes was estimated with the delta delta Ct method [33] using two reference genes: elongation factor-1a (GenBank KT185020) and ribosomal protein subunit 18 (GenBank KT279693) [34].

### 2.5. Statistical Analyses

All data analyses were carried out with JMP Version 12 (SAS Institute Inc., Cary, NC, USA) and GraphPad Prism 7 Software (GraphPad Software, San Diego, CA, USA). Survival test was determined using Kaplan-Meier survival curves and log-rank test. The *P*-values of comparisons between the psyllids feeding on diets lacking ConA or with ConA (“Lso-free” vs. “Lso-free + ConA”, “LsoA” vs. “LsoA + ConA”, and “LsoB” vs. “LsoB + ConA”) were calculated. The test among the six groups was conducted as well. In addition, the mortality among Lso-free, LsoA-, and LsoB-infected psyllids feeding on ConA-containing diets at each day were compared using one-way ANOVA with Tukey’s *post hoc* test. Effects of ConA on caspase gene expression were determined with Student’s *t*-tests.

## 3. Results

### 3.1. Mortality of Potato Psyllids Following ConA Treatment

The mortality of Lso-free, LsoA-, or LsoB-infected potato psyllids was monitored following feeding on diets containing ConA. The results showed that ConA significantly increased psyllid mortality when compared to the control diets lacking ConA whether psyllids were Lso-free, LsoA-, or LsoB-infected (log-rank test: *p* < 0.0001 for all the comparisons) (Figure 1b). In particular, after two days of feeding on the ConA-containing diets, the number of dead LsoA-infected psyllids was significantly higher than Lso-free psyllids (*F*_2,6_ = 5.182, *p* < 0.05), however, there was no significant difference in the survival rate among Lso-free and Lso-infected psyllids feeding on ConA after three days (*p* > 0.05). By day six, none of the potato psyllids were alive in the ConA-containing treatments, while around 90% of the Lso-free, LsoA- or LsoB-infected psyllids were still alive in the control treatment (Figure 1b).

### 3.2. Nuclear Architecture and TUNEL Assay

**Figure 2:**
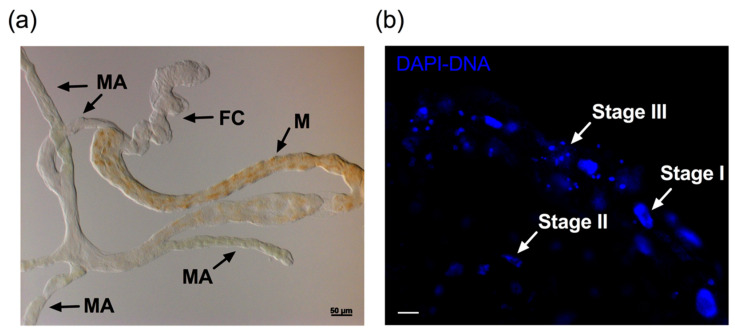
Nuclear architecture changes after ConA feeding. (**a**) Light micrograph of the potato psyllid alimentary canal showing characteristic structures. FC: filter chamber, M: midgut, MA: midgut appendages. (**b**) The nuclear morphology (blue, DAPI staining) in the gut of potato psyllids after feeding in sucrose diets containing ConA for 72 h. Stages I, II, and III represent the three stages of apoptotic nuclei changes. Scale bar is 20 μm.

**Figure 3:**
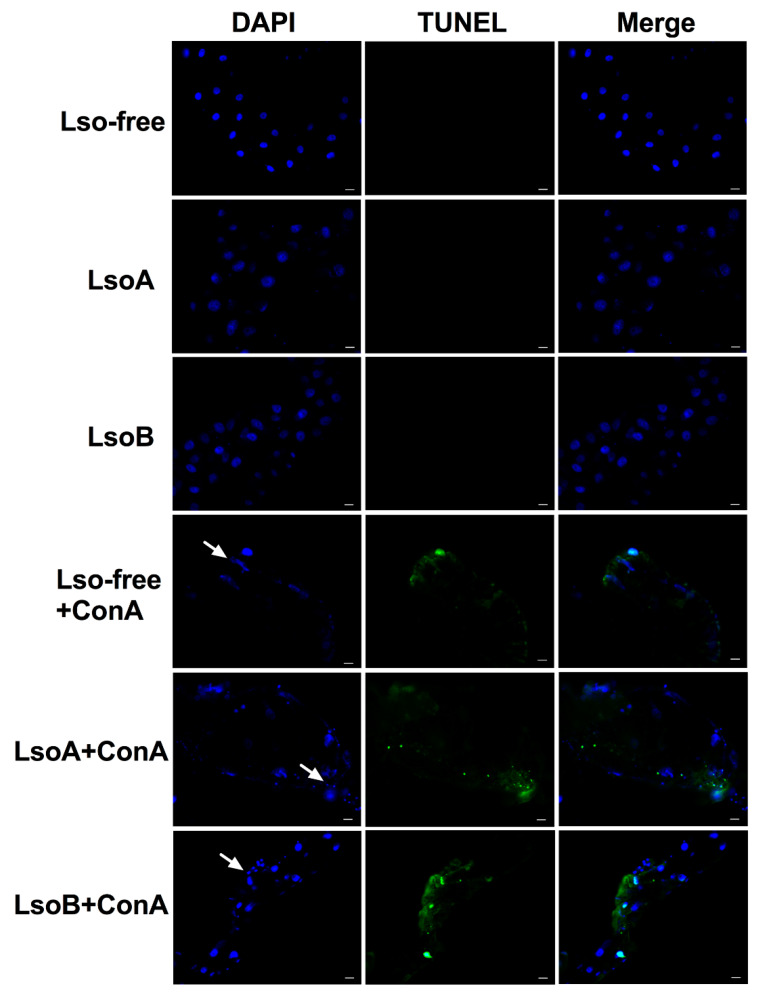
ConA induced apoptosis in the gut of Lso-free, LsoA-, and LsoB-infected potato psyllid. The tissues were stained using TUNEL assays to detect the apoptotic signals (green) and counterstained with DAPI to show the nuclei (blue) of the gut cells. The white arrows indicate the apoptotic nuclei. Scale bar is 20 μm.

The midgut epithelial cell nuclei of ConA-treated psyllids showed a typical pattern of changes observed in apoptotic cell nuclei. These changes mainly occurred in the midgut (Figure 2a,b). Figure 2b shows the three representative stages of apoptotic changes of nuclei in potato psyllid midgut cells after ConA feeding as described by Kihlmark*,* et al. [35]. These morphological events were observed in the guts of the Lso-free, LsoA-, or LsoB-infected potato psyllids exposed to ConA (Figure 3 and Figure S1). Specifically, the gut cells of insects fed with ConA (Figure 2 and Figure 3) showed several nuclei that seemed to be in the earliest stage of apoptosis, Stage I, displaying a punctate distribution. A few nuclei were condensed and collapsed, showing the signs of stage II. In stage III, many nuclei formed grape-shaped apoptotic bodies and exhibited pyknosis and/or karyorrhexis, and most of the nuclei had lost their elliptical round shape. In contrast, midgut cell nuclei from the psyllids feeding on the control diet appeared regularly dispersed in the cells and their shape, size, and DAPI staining were uniform (Figure 3).

Concomitantly, no signal was detected in the midgut of psyllids from the control treatment by TUNEL assay. In contrast, several cell nuclei from Lso-free, LsoA- or LsoB-infected psyllid midguts showed signals of DNA fragmentation following ConA treatment. Importantly, the signal of DNA fragmentation co-localized with the broken nuclei as observed in the merged views (Figure 3 and Figure S1).

### 3.3. Expression of Caspase Genes

**Figure 4:**
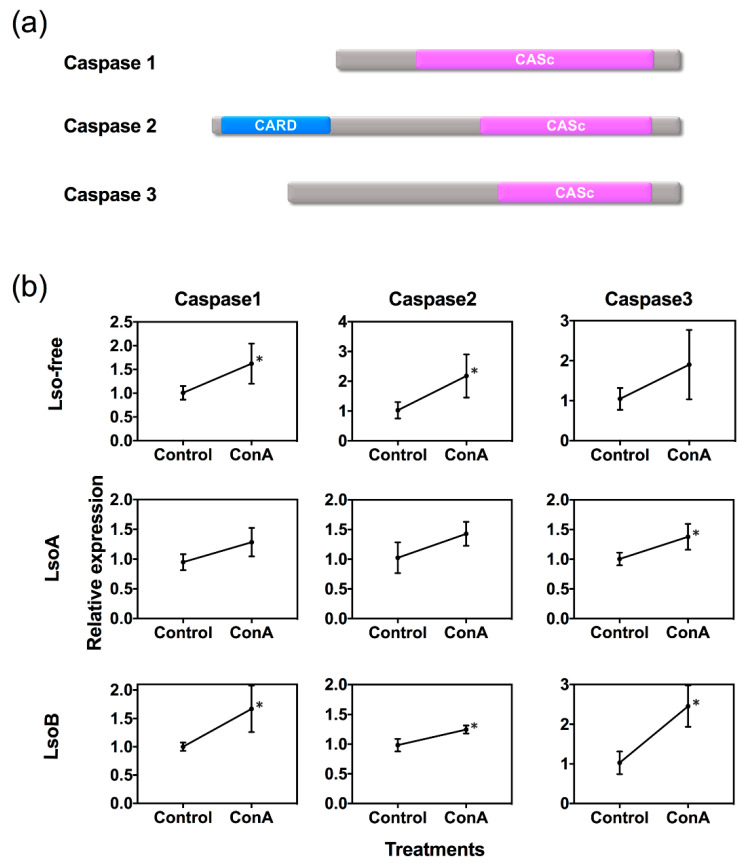
Gene structure and expression profiles of caspases. (**a**) Gene structure of the three caspases. CARD indicated caspase recruitment domain. CASc domain comprised of a large and a small subunit. (**b**) Significant upregulation of caspase 1, caspase 2 and caspase 3 genes in the guts of Lso-free, LsoA-, and LsoB-infected potato psyllids after 72 h exposure to ConA. * indicates *p* < 0.05.

In total, three caspase genes (caspase 1–3) were identified in the psyllid transcriptome dataset [31]. The domains of caspases were identified in our previous study [32]. Caspase 1 is a putative effector caspase with a short pro-domain, while caspase 2 is a putative initiator caspase with a caspase recruitment domain (CARD). Caspase 3 is another putative effector with its serine- and threonine-(ST-) rich pro-domain (Figure 4a). Our results showed that the three caspases exhibited upregulation at the transcriptional level in the gut of Lso-free or Lso-infected psyllid after 72 h of exposure to ConA (Figure 4b). Caspases 1 and 2 were significantly upregulated in the gut of Lso-free psyllids after 72 h of exposure to ConA. All caspases (1, 2 and 3) were significantly upregulated in the gut of LsoB-infected psyllids exposed to ConA, while, only caspase 3 was significantly upregulated in the gut of LsoA-infected psyllids exposed to ConA. 

## 4. Discussion

Lectins have received much attention for their remarkable pesticidal activities and their potential for pest control applications (e.g., insect-resistant transgenic crops) [36,37,38]. In particular, lectins comprise the best available toxins and display a wide array of molecular targets for the control of hemipteran pests [39,40,41]. In the present study, we found that ConA possesses high toxicity against potato psyllids and caused significant mortality. Indeed, this is consistent with other artificial diet studies, which showed that ConA has insecticidal activity, particularly towards hemipteran pests [11,12,42]. Interestingly, ConA caused greater mortality in LsoA-infected psyllids after two days of feeding when compared to the effects on Lso-free psyllids. It is most likely that ConA toxicity coupled with Lso infection pose a dual detrimental effect on psyllids because Lso has negative effects on the potato psyllid physiology; for example, Lso infection results in decreased psyllid oviposition and nymphal survival [28,43]. Recently, a study in the interaction of Asian citrus psyllid—‘*Ca*. L. asiaticus’ (CLas) determined that the presence of CLas-induced apoptosis in adult psyllid midgut cells [44]. However, this phenomenon does not appear to occur in the potato psyllid in response to Lso [32]. One hypothesis to explain the absence of detectable apoptosis in the gut of Lso-infected adults is that the Lso-induced intracellular immune response did not reach or exceed the threshold to trigger an intracellular apoptotic immune reaction [45]. However, higher mortality was observed for the LsoA-infected psyllids than the uninfected psyllids after two days of feeding on the ConA-containing diets. Therefore, it is possible that intense death-inducing stimuli might have resulted from the combination of Lso infection and ConA treatment [45], reaching the threshold of cell death and resulting in greater mortality for Lso-infected psyllids in less amount of time. In addition, although intracellular bacteria are known to manipulate (usually inhibit) insect host apoptotic responses [27], it appears that the presence of Lso in potato psyllids is not able to disrupt the ConA-induced apoptosis. Therefore, ConA is a promising tool as a novel strategy to control Lso-free and Lso-infected psyllids. 

A limitation of the current study is that a no-diet control was not included. While in our experience low mortality is registered when potato psyllids adults are kept for two or three days without any diet, other studies have shown that hemipteran first instar nymphs die quickly in the absence of diet [46]. Future experiments should include the no-diet control to confirm that ConA has a toxic effect rather than preventing or reducing feeding, and if psyllid mortality is the result of toxicity or combination of both toxicity and reduced feeding. Although the insecticidal properties of ConA are well studied, the mechanisms or molecular bases of this effect remain largely unknown. Lectins are known to cause a range of effects on mammalian cells including agglutination, induction of mitosis, interference with general metabolism, impairment of membrane transport systems or increased membrane permeability to intracellular proteins [47,48]. In insects, both ultrastructural and immunolocalization studies showed that lectins could bind to glycosylated targets or receptors in the gut epithelium cells [10,49], and this binding can cause severe cellular swelling of the midgut epithelium cells [10]. In the present study, we observed ConA-induced morphological changes of the nucleus architecture in the midgut epithelium cells of potato psyllids. These morphological changes in the epithelium cells are potential signs of apoptosis. Indeed, the three stages of nuclei disruption from cells undergoing apoptosis were identified in the psyllid midgut cells following ConA ingestion [35]. In addition, apoptosis is characterized by producing characteristic DNA fragmentation, which is a hallmark of apoptosis that distinguishes apoptosis from necrosis [50]. Signals of DNA fragmentation based on TUNEL assay were only identified in the midgut cells of ConA-treated psyllids. Furthermore, the TUNEL signals were simultaneously detected with the broken nuclei in the midgut epithelium, which further confirmed that apoptosis was induced in the psyllid midguts after feeding ConA-containing diets. We hypothesize that the abnormal nuclear architecture caused by apoptosis may result in the disruption of the gut epithelium homeostasis with subsequent impairment of the insect physiology. Indeed, it has been shown that the cytotoxicity of lectins could be mediated via induction of apoptosis [11,12,51] as was determined in grain aphids fed with ConA, resulting in apoptosis and subsequent death [11].

Apoptosis is known to play a major role in the development and/or stress responses of organisms [52,53,54]. In insects, apoptosis is a vital defense mechanism against pathogens [55,56], and it can affect the efficiency of pathogen transmission [57]. Caspases, a group of cysteine proteases, are the central components of the apoptotic response that initiate and execute the apoptotic cell death [58,59]. Although caspases are well characterized in many organisms, little is known about insect caspases compared to those in mammals, and especially hemipteran caspases. Three caspase genes were identified from the published potato psyllid transcriptome datasets, which included one putative initiator caspase (caspase 2) and two putative effectors (caspases 1 and 3). The three caspases were upregulated at the transcriptional level in the guts of psyllids from the ConA treatment, which represents further evidence that ConA induces apoptosis in the psyllid midgut cells. Therefore, the caspase-dependent pathway appears to be one of the responses induced in the potato psyllid midgut after feeding on ConA-containing diets. However, differences in the regulation of those caspases among Lso-free, LsoA-, and LsoB-infected insects were observed. It cannot be excluded that those differences are the result of differences in the mechanisms or proteins used by different Lso haplotypes to manipulate the insect vector defenses.

The observation of apoptosis may imply that binding to midgut epithelial cells could be a causative factor in the toxicity of ConA. In detail, the changes inflicted by ConA-binding to gut surface receptors could result in changes in metabolism and cell function in the epithelium, which in turn may lead to high mortality [18]. Therefore, the insecticidal activity of lectins is probably associated with the sugar-binding capacity of these proteins [12]. However, not all the lectins can bind to the midgut or even the whole digestive tract, causing morphological changes in the cells as well as increased secretion and detachment of the apical membrane. For example, no lysis of epithelium cells was seen in another hemipteran insect, *Lygus hesperus*, following PHA treatment [60].

## 5. Conclusions

In summary, we demonstrated that ConA has a significant deleterious effect on potato psyllid survival. A key mechanism underlying this detrimental effect was associated with apoptosis in midgut epithelial cells. While not tested, binding of ConA to the midgut epithelium cells could result in changes in the gut nuclei morphology and function, or even the metabolism as shown in other species. In the future, efforts should be aimed at identifying the specific targets or receptors in psyllid midgut. Overall, this study helps us better understand the mode of action of ConA at the cell and tissue levels, which could work as a prerequisite for its use in transgenic crops.

## Supplementary Materials

The following are available online at https://www.mdpi.com/2075-4450/11/4/243/s1, Figure S1: ConA induced apoptosis in the gut of Lso-free, LsoA-, and LsoB-infected potato psyllid. The tissues were stained using TUNEL assays to detect the apoptotic signals (green) and counterstained with DAPI to show the nuclei (blue) of the gut cells. Scale bar is 20 μm, Table S1: Primers for bioinformatics validation and gene expression analyses by qPCR of caspase genes.
