# Supplementary material for: Concanavalin A Toxicity Towards Potato Psyllid and Apoptosis Induction in Midgut Cells
Source: Insects. 2020 Apr 14;11(4):243. doi: 10.3390/insects11040243 (PMC7240484; doi:10.3390/insects11040243)
Supplement: Supplementary file 1 [file insects-11-00243-s001.pdf]

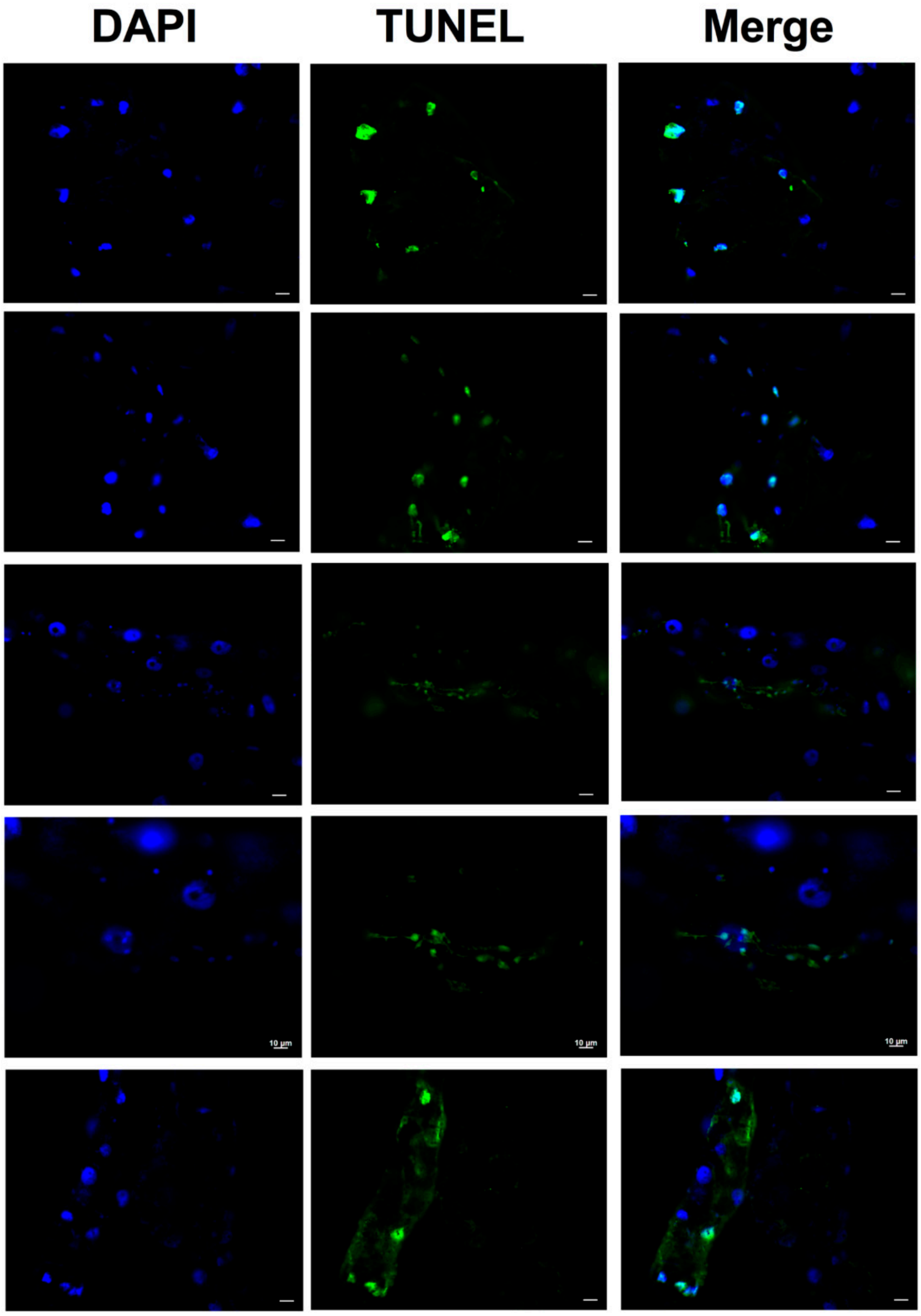

Figure S1. ConA induced apoptosis in the gut of Lso-free, LsoA- and LsoB-infected potato psyllid. The tissues were stained using TUNEL assays to detect the apoptotic signals (green) and counterstained with DAPI to show the nuclei (blue) of the gut cells. Scale bar is 20 μm.

Table S1. Primers for bioinformatics validation and gene expression analyses by qPCR of caspase genes.

Gene Name	Code	Primers (5'-3') for Bioinformatics Validation	qPCR Primers (5'–3')
Caspase-1 isoform X1	Caspase1	F: AAAATCGTCTCCAGGCTTCA	F: CAAGGAGATGGTCTGGATGG
		R: TCAGAGTGGTCAAGTTCCTTTC	R: ATGAGGAAGTCAGCGTGGAG
Caspase-2-like	Caspase2	F: TTTGATGCTTTGTGGGA	F: ATGTCCCCAGCAATGGTATC
		R: AACTTGGGAATGTTGGC	R: CACAGGGTGTTGACTTCTTCA
Caspase-3-like	Caspase3	F: GTAAGCAACAATACGACACC	F: AAGCTGGATGGTGGAGTACG
		R: CATAGGAGGGGATCTTGTAG	R: CAGCATAGGAGGGGATCTTG
